# A common source of attention for auditory and visual tracking

**DOI:** 10.3758/s13414-018-1524-9

**Published:** 2018-05-01

**Authors:** Daryl Fougnie, Jurnell Cockhren, René Marois

**Affiliations:** 1 Department of Psychology, New York University Abu Dhabi, Abu Dhabi, United Arab Emirates; 2 Department of Psychology, Vanderbilt University, Nashville, TN USA

**Keywords:** Divided attention, Inattention, Dual-task performance

## Abstract

Tasks that require tracking visual information reveal the severe limitations of our capacity to attend to multiple objects that vary in time and space. Although these limitations have been extensively characterized in the visual domain, very little is known about tracking information in other sensory domains. Does tracking auditory information exhibit characteristics similar to those of tracking visual information, and to what extent do these two tracking tasks draw on the same attention resources? We addressed these questions by asking participants to perform either single or dual tracking tasks from the same (visual–visual) or different (visual–auditory) perceptual modalities, with the difficulty of the tracking tasks being manipulated across trials. The results revealed that performing two concurrent tracking tasks, whether they were in the same or different modalities, affected tracking performance as compared to performing each task alone (concurrence costs). Moreover, increasing task difficulty also led to increased costs in both the single-task and dual-task conditions (load-dependent costs). The comparison of concurrence costs between visual–visual and visual–auditory dual-task performance revealed slightly greater interference when two visual tracking tasks were paired. Interestingly, however, increasing task difficulty led to equivalent costs for visual–visual and visual–auditory pairings. We concluded that visual and auditory tracking draw largely, though not exclusively, on common central attentional resources.

**Electronic supplementary material:**

The online version of this article (10.3758/s13414-018-1524-9) contains supplementary material, which is available to authorized users.

Our sensory environment contains an abundance of information—far more than we can attend to at any given time. Such attentional limitations are easily evidenced by the multiple object tracking (MOT) paradigm. In this paradigm (Pylyshyn & Storm, 1988), participants are required to attend to and track a subset of discs as they move about unpredictably among like distracter discs. To differentiate the targets from distractors, participants must follow the spatiotemporal path of each target disc. Under such conditions, only three to five targets can be successfully tracked (Pylyshyn & Storm, 1988; Scholl, 2001; Sears & Pylyshyn, 2000). Similar limits are also observed in tracking tasks that use nonspatial visual features such as color (Fougnie & Marois, 2006).

Although the characteristics of attentional limitations in visual tracking tasks have been well studied (Allen, McGeorge, Pearson, & Milne, 2006; Alvarez & Cavanagh, 2005; Alvarez & Franconeri, 2007; Fehd & Seiffert, 2008; Pylyshyn, 1994, 2001; Pylyshyn & Storm, 1988; Scholl, 2001, 2009; Shim, Alvarez, & Jiang, 2008; Tombu & Seiffert, 2008; Wolfe, Place, & Horowitz, 2007), less attention has been paid to the exploration of tracking in other sensory modalities, such as tracking an auditory stream of tones presented among distractor auditory streams. Hence, it is not known whether similar attentional constraints operate in other sensory domains than vision, and whether tracking information in any sensory domain draws on sensory-specific or central, amodal resources. Such central, amodal resources would be revealed if tracking in one modality—say visual—interfered with tracking in another modality—such as auditory.

Although no previous study has specifically investigated dual-task interference between auditory and visual tracking, there has been extensive work examining whether processing capacities in other attention tasks are modality-general or modality-specific (Alais, Morrone, & Burr, 2006; Cowan, 1995; Duncan, Martens, & Ward, 1997; Martens, Kandula, & Duncan, 2010; Spence & Driver, 1996; Tombu & Seiffert, 2008). Unfortunately, little consensus has emerged from this work. Several studies have shown convergence between auditory and visual information in the attentional selection of perceptual inputs (Eimer & Schroger, 1998; Ghazanfar & Schroeder, 2006; Hocherman, Benson, Goldstein, Heffner, & Hienz, 1976; Kayser, Petkov, Augath, & Logothetis, 2007; Spence & Driver, 1996, 1997; Spence, Nicholls, & Driver, 2001). For example, valid exogenous and endogenous spatial cues from one modality (e.g., auditory) facilitate responses to stimuli that occur in a different modality (e.g., vision) at the cued location. However, although such paradigms demonstrate that selection of a particular region of space has cross-modal benefits, they are not designed to reveal sources of attentional limitations, and therefore cannot speak to the modal nature of such capacity limited resources. On the other hand, tasks that require monitoring distinct, competing streams of visual and/or auditory information to identify or detect targets have generally pointed to independent sources of visual and auditory perceptual attention (Alais et al., 2006; Duncan et al., 1997; Lee, Koch, & Braun, 1999). Specifically, participants who are monitoring two sources from the same modality (i.e., the visual modality) can only report properties of one stimulus accurately (Bonnel & Miller, 1994; Lee et al., 1999; Norman & Bobrow, 1975; Sperling & Dosher, 1986), but they show no such costs for dividing attention across modalities (e.g., the auditory and visual modalities; Alais et al., 2006; Duncan et al., 1997; Larsen, McIlhagga, Baert, & Bundesen, 2003; Martens, Johnson, Bolle, & Borst, 2009; Martens et al., 2010; Soto-Faraco & Spence, 2002; Treisman & Davies, 1973). Such results can be taken as support of independent sources of attention for vision and audition (Alais et al., 2006).

Although these target detection and identification tasks do require selection and the processing of task-relevant information, they lack an important aspect of attention—the continuous updating of attended information (Allport, 1993; Blaser, Pylyshyn, & Holcombe, 2000; Chun, Golomb, & Turk-Browne, 2011; Egeth & Yantis, 1997; Huang & Pashler, 2007; Kane & Engle, 2002; Serences et al., 2005; Wolfe et al., 2007). The target detection/identification studies typically require participants to maintain focus on a particular stimulus or stimulus dimension over long durations, thus placing very little load on online attentional control mechanisms. Instead participants may rely solely on maintaining the same attentional task set and target templates to perform the monitoring task (Duncan & Humphreys, 1989), perhaps by optimally configuring the sensory system prior to stimulus onset. For example, if a task requires detecting whether a line of a certain orientation is present among distractors, the gain of neurons that code for that orientation can be increased, raising the likelihood that those neurons will fire with a weak stimulus input (Maunsell & Treue, 2006; Treue & Martínez-Trujillo, 1999). Tasks in which the differentiation of target from distractor stimuli is based on time-invariant feature(s) may not be optimal for testing whether there are independent sources of attentional control for auditory and visual attention, because these tasks make few ongoing attentional demands.

In contrast, attentive tracking tasks place demands on both attentional selection (Pylyshyn & Storm, 1988) and control (Alvarez & Cavanagh, 2005; Blaser et al., 2000) since participants not only have to select the targets from the distractors, but also to repeatedly update the selection settings as the feature value(s) that differentiate the target from the distractor changes over the course of tracking. If an attentional template were to be involved to perform such a task, that template would need to be constantly updated. Because of the attentional control demands of attentive tracking tasks, and because attentional control is thought to be a central, executive function (Pashler, 1998), we propose that tracking tasks will interfere at central sources of processing regardless of the modality. Although this has not been directly tested, there is considerable evidence that tracking tasks interfere with a wide variety of attention-demanding tasks (Allen et al., 2006; Alvarez, Horowitz, Arsenio, DiMase, & Wolfe, 2005; Fougnie & Marois, 2006, 2009; Kunar, Carter, Cohen, & Horowitz, 2008; Tombu & Seiffert, 2008). Of particular importance is the evidence that tracking can disrupt tasks that involve word generation and response selection (Kunar et al., 2008; Tombu & Seiffert, 2008). These are high-level processes that are believed to draw on central sources of attention and interfere with one another even if they draw on distinct modalities (Arnell & Jolicœur, 1999; Bonnel & Hafter, 1998; Jolicœur, 1999; Potter, Chun, Banks, & Muckenhoupt, 1998; but see Tulving & Lindsay, 1967). It is unclear, however, the extent to which two tracking tasks would interfere with one other: Would the source of interference primarily originate from having to perform two tasks concurrently? Would interference be proportional to tracking demands? And to what extent would these sources of interference be amodal? In sum, is there anything inherently visual about the source of the attention limit revealed by a multiple object tracking task?

Here we asked whether the degree to which tracking visual and auditory information draws on a shared or on distinct sources of attention control. The basic methodology consisted in comparing the interference between tasks that either differed in modality (i.e., visual and auditory tasks) or shared a modality (i.e., two visual tasks). Two distinct visual tracking tasks and an auditory task were used for these experiments. In one of the visual tracking tasks—the dot task—participants were required to track a target dot while ignoring a featurally similar distractor dot by following the target’s spatiotemporal position. Such tracking tasks have been well studied and have demonstrated limits in visuospatial attention (Alvarez & Cavanagh, 2005; Oksama & Hyönä, 2004; Scholl, 2009; Sears & Pylyshyn, 2000). In a second visual tracking task (Gabor task), participants tracked one of two spatially overlapping Gabors by attending to the target Gabor’s color, orientation, and spatial frequency as both the target and distractor Gabors’ feature properties changed over time (Blaser et al., 2000; see Neisser & Becklen, 1975, for a similar task). The auditory task employed in the present study required participants to track one of two auditory streams as the sounds changed in pitch and stereo position (i.e., left or right stereo space). Studies of auditory perceptual segregation have demonstrated that it is possible to selectively attend to one of two concurrently presented auditory streams if the streams are presented as a series of alternating tones, a phenomenon termed the “streaming effect” (Bregman, 1990; Bregman & Campbell, 1971). Previous studies have used this phenomenon to understand which elements of complex sound sequences can be attended (Brochard, Drake, Botte, & McAdams, 1999; Large, Fink, & Kelso, 2002). Our study used the auditory streaming effect as a means to study auditory attentive tracking by presenting two competing streams and requiring participants to selectively focus on one stream.

The amount of overlap in processing shared by two tracking tasks can be inferred from the costs of having to perform both tasks concurrently, relative to performing either task in isolation. Here we measured the extent of these dual-task costs for task pairings that differed in the modality of the to-be-tracked stimulus (i.e., auditory and visual), and compared how the extent of these costs compared to the costs for task pairings that shared a modality (i.e., two visual tasks). If the attentional resource that allows us to track information over time is purely modality-dependent, then there should be no observable dual-task costs when the tracking tasks differ in perceptual modality. On the other hand, if the ability to track items over time is purely modality-independent, than the costs incurred by having to perform two tracking tasks rather than one would be equivalent for the different task pairings. To preview our findings, we found substantial interference when participants tracked auditory and visual information concurrently, and that visual and auditory tracking draw largely, though not exclusively, on common attentional resources.

## General method

Here we measured the costs for performing two tracking tasks for tasks that differed in modality (visual and auditory, Exp. 1) and for tasks that shared a modality (two visual tasks, Exp. 2). Each trial consisted of the presentation of two tracking displays. On some trials participants were instructed to ignore one of the tracking tasks, allowing us to assess performance for single- and dual-task trials. We manipulated the difficulty of each tracking task (each task had an easy and hard difficulty level) to show that interference was not just due to difficulty in coordinating and preparing for two tasks.

### Tracking outline

The tracking tasks had encoding, tracking, and response phases. Note that if a trial involved two tracking tasks, the phases completely overlapped for the two tasks. A trial began with target encoding (3,000 ms). Participants were presented with a target for each of the two tasks and were instructed to encode both targets. Targets remained onscreen for the entire duration of the target encoding period. We presented two targets on all trials (including single-task trials) so that encoding demands were similar for single- and dual-task conditions. The task cue (2,000 ms) instructed participants which target(s) they should follow during the tracking interval. Following the 2,000 ms of cue presentation, the cue was removed, and the stimuli remained for 2,000 ms. This 2,000-ms postcue phase gave participants more than adequate time to adjust their task set to attend to the task-relevant target(s). Following the postcue period, a single distractor that was featurally similar to the target was added to each task. The ensuing distractor onset phase (1,000 ms) gave participants time to adjust to *selectively* attending to the target(s) before the onset of tracking.

During the tracking interval (8,000 ms), the target and distractor for each task continually changed in the task-relevant features (see the individual tracking task methods). To differentiate target from distractor, participants needed to constantly attend to the target since the feature values that allow the target to be distinguished from the distractor changed over time. Note that during the tracking phase the stimuli for both tasks were presented, even during single-task conditions. Thus, differences in performance between single- and dual-task conditions cannot be explained by differences in sensory stimulation during attentive tracking.

### Collecting responses

To test whether participants successfully tracked the target in the cued task(s), only one of the two stimuli remained on at the end of the tracking interval, and participants were to indicate whether the remaining stimulus was a target or a distractor (with each possibility equally likely). For example, in a dual-task trial involving an auditory and visual tracking task, the probe may consist of either the presentation of the visual target, visual distractor, auditory target, or auditory distractor. In single-task trials, the probe was always for the cued task. Participants were instructed to indicate whether the probe item was a target or distractor by selecting one of two buttons with their right hand. Responses were unspeeded, and accuracy was stressed. Trials were self-paced and accuracy feedback was provided after each response.

### Task difficulty manipulation

There were two difficulty levels—easy and hard—for each task. The rate of feature change in the tasks was adjusted in order to achieve a certain percent correct in the single-task conditions. The difficulty adjustments were made at the end of each block. For the easy condition, whenever participants made at least one error in the single-task trials of the previous block, the subsequent block had a slower rate of feature change. For the hard condition, if performance was above/below 75% in the previous block the rate of feature changes in the subsequent block was made to be faster/slower. This difficulty adjustment was performed to minimize differences in task demand across tasks and across individual differences in task competence. For each participant, performance in the easy single-task conditions should approach 100% and performance in the difficult single-task conditions should approximate 75%.

### Trial structure

Each participant completed two single-task blocks, followed by five intermixed blocks containing both single- and dual-task trials. The first two single-task blocks were intended for adjusting the task difficulty and were not included in the main data analysis. The intermixed blocks consisted of eight trials of each single-task condition and four trials of each of the eight dual-task conditions, for a total of 64 trials. Thus, single- and dual-task trials occurred equally often in intermixed blocks. Participants completed the seven blocks across two days, with no more than two days’ separation.

### Bonus pay

To motivate participants to perform at their best, we provided bonus pay on each day ranging between $0–$10. The payout was a combination of $0–$5, based on task difficulty setting at the end of the experiment (single-task bonus), and $0–$5, based on minimizing dual-task costs (dual-task bonus).

#### Single-task bonus

For a participant to receive no single-task bonus pay, tracking difficulty would have to decrease after each of the seven blocks of trials. For a participant to receive the maximum ($5) bonus pay, tracking difficulty would have to increase after each block. The exact monetary amount was determined by comparing the number of difficulty levels (averaged across tasks and easy/hard conditions) achieved by the end of the experiment with the highest achievable difficulty level. Concretely, if a participant’s average final difficultly level was 80% of the highest achievable value, they would receive 80%, or $4, of the maximum single-task bonus ($5).

#### Dual-task bonus

The bonus for dual-task performance was determined by how that performance fared relative to single-task performance (averaged across tasks and conditions). If a participant could not perform in dual-tasks better than chance level (50%), they earned no bonus pay. If a participant performed at or above single-task performance level in dual-task trials, they received the maximum ($5) bonus pay for dual-task trials. Bonus pay for values in between was set by the relative position of dual-task accuracy within the range of chance level and single-task performance. For example, if a participant performed at 100% accuracy in single-task trials and at 70% accuracy in dual-task trials, since dual-task performance was 40% of the distance between chance and the single-task performance, they would receive 40%, or $2, of the maximum dual-task bonus ($5).

Participants were not told the details of the bonus pay calculations. They were simply told that bonus pay was linked to their overall task performance and that they should try to perform well on all trials.

### Visual Gabor task

In the Gabor-tracking task, participants were asked to track which of two overlapping Gabors is a target. When two Gabors are presented in the same spatial location, but alternate at a fast rate, participants can perceive and segregate both Gabors (Blaser et al., 2000). In our task we alternated between the target and distractor Gabor presentation every 10 ms. The Gabor patches (1.5° × 1.5°) were defined by color, orientation, and spatial frequency. Attending to only one feature would be insufficient to differentiate the two Gabors, since, at any point the two Gabors could share a value in one feature. However, the Gabors were never the same value in two or more features at any given time. A previous study showed that participants can selectively track multiple features of a single Gabor (Blaser et al., 2000).

#### Stimuli

Color values for the two Gabors were drawn from a circle in the CIE L*a*b* color space (centered at L = 54, a = 18, b = – 8, with a radius of 59). The spatial frequency of the two Gabors was randomly assigned within the range of 0.35°–0.85° of visual angle/period (angle per period, or APP). The initial value of the two Gabors were chosen to differ by at least 60° in orientation, 60° in L*a*b* color space, and 0.1° APP.

#### Rate of feature change

In the easy task condition, color and orientation initially changed at a rate of 1.25° (in color or orientation space) per 20 ms (i.e., every other refresh), whereas spatial frequency changed at a rate of 0.01° APP. In the hard task condition, color and orientation initially changed at a rate of 1.75°/20 ms, whereas spatial frequency changed at a rate of 0.014° APP. These initial values were adjusted over the course of the experiment to achieve a desired level of performance for each condition. To improve performance, the color and orientation change rates decreased by 0.25°, and the spatial frequency change rate decreased by 0.002° APP. To reduce performance, the rates of change for color and orientation increased by 0.25°, and the spatial frequency change rate increased by 0.002° APP. The minimum change rates were 0.25° for color and orientation and 0.002° APP for spatial frequency. For four participants the initial difficulty settings were different (1.0° for the easy condition; 1.25° for the hard condition), but the final difficulty levels for these participants were similar to the group average. The direction of change was set randomly per feature per Gabor and had a 10% chance of changing direction at every refresh (set independently for each feature).

#### Task cue

**Fig. 1:**
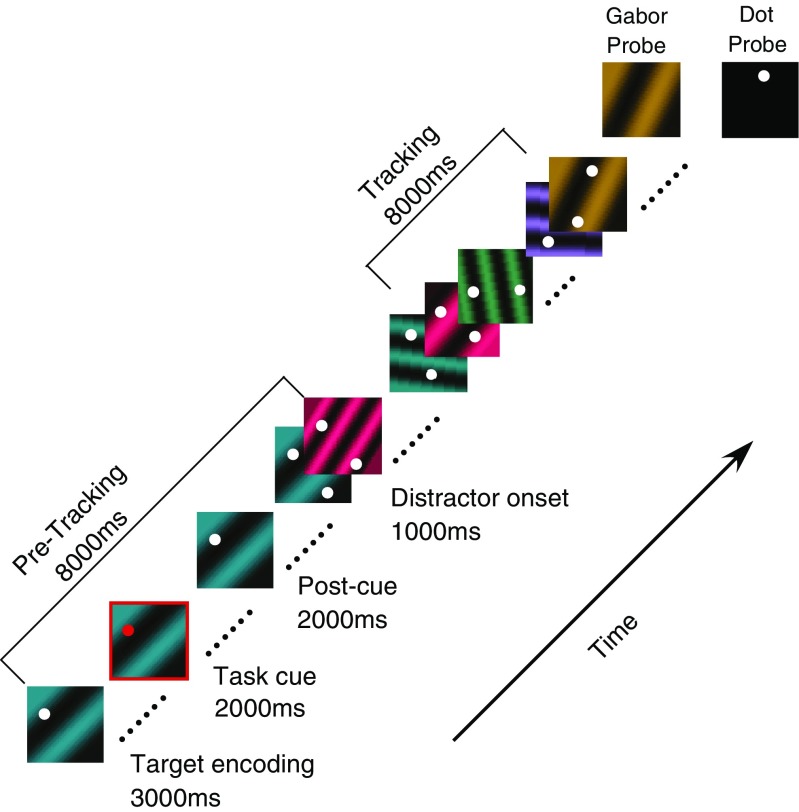
Timeline for a dual-task trial involving the Gabor- and dot-tracking tasks. During the pretracking phase (8,000 ms), participants were shown a white dot overlaid over a colored Gabor. A cue appeared (3,000 ms), indicating whether the task would involve dot tracking, Gabor tracking, or both. The cue was onscreen for 2,000 ms, and then, a further 2,000 ms after it had disappeared, the distractor stimuli appeared (a second dot and second Gabor). The Gabors alternated every screen refresh for 1,000 ms, allowing both to be visible. The dots and Gabors then changed in featural values over an 8,000-ms tracking interval. Participants had to follow the identity of the target(s) stimuli and indicate whether or not a single probe was the target (equally likely). In dual-tracking trials, the probed task (Gabor or dot task) was selected at random

The task cue to signal participants to attend to the Gabor task was a red frame (0.08° thick) surrounding the target Gabor. This task cue appeared for 2,000 ms during the pretracking phase (see Fig. 1).

### Auditory tone task

In the tone-tracking task, participants were asked to track which of two auditory streams was the target stream. When a series of brief tones of two distinct pitches are alternately presented at a high rate, participants perceive the two tones as two distinct “auditory streams” segregated by pitch (Bregman & Campbell, 1971). In the present study we used this auditory phenomenon to create an auditory tracking task in which participants tracked a stream of target tones that could be differentiated from the distractor stream by its distinct (and unpredictable) trajectory in pitch and stereo space (left, center, or right).

To describe this task, it is useful to first clarify some terminology. The stimuli consisted of alternating target and distractor tones (a target tone was always followed by a distractor tone, and vice versa). The tones were brief bits of sound at a constant pitch. During the tracking phase, each stream would change its pitch and stereo position every six tones. We use the term transition cycle to define this six-tone period.

#### Stimuli

At the start of each trial, the target and distractor streams were assigned initial pitch values between 220 and 1540 Hz in steps of 120 Hz, with the restriction that the two starting pitch values differed by at least 360 Hz. Additionally, the distractor and target were assigned distinct positions in stereo space (left, right, or center). The left and right position of stereo space corresponded to the sound being played to the left or right ear, respectively, with no sound played to the other ear. The center position of stereo space involved sound presentation to both ears at 50% intensity.

During the tracking phase, the target and distractor streams would gradually change to a new pitch every six tones (transition cycle). For example, a target stream changing from 220 to 580 Hz would change pitch in six equally spaced steps; 280, 340, 400, 460, 520, and 580 Hz. At the same time a distractor stream might change pitch from 700 to 220 Hz. For each transition, the pitch of each stream changed by at least 120 Hz and, after completing the transition, respected the 360-Hz minimum pitch difference between the target and distractor streams.

The streams would also change in stereo position during tracking. Since stereo position was assigned independently for the target and distractor streams, the two streams could overlap. However, when the two streams crossed pitch (e.g., the target was initially a higher pitch than the distractor, but then become lower), the two streams were not allowed to share a stereo position. The exception was necessary because the streaming effect requires perceptual segregation cues (Bregman & Campbell, 1971), and when the two pitch values crossed, location differences were needed in order to maintain segregation. To prevent participants from relying on categorical labels (e.g., “left” or “high”), we ensured that trials contained at least one pitch crossing (average of 3.44 crosses per trial) and that streams changed in stereo position at least once per trial (average of 4.08 stereo position changes per trial).

#### Rate of feature change

At the start of the experiment, the easy condition had nine transition cycles. Whenever participants made an error on single-task trials, the number of transition cycles decreased by one for subsequent blocks. The hard condition started with 12 transition cycles. Whenever accuracy was above/below 75%, the number of transition cycles increased/decreased for subsequent blocks. The alternation rate (time between successive tones) was determined by the number of transition cycles. The tracking interval was fixed at 8,000 ms, and each transition cycle contained 12 tones (six each for the target and distractor streams). For example, if there were eight transition cycles, each transition would take 1,000 ms (8,000/8), and the alternation rate would be 83.3 ms (1,000/12).

#### Task cue

To cue that the tone task was the relevant task, the intermittent target tone stream was replaced by a long steady beep of the same pitch, intensity, and position in stereo space as the target. This beep lasted the duration of the task cue phase (2,000 ms).

### Visual dot task

The dot task is a variant of the multiple-object tracking task (Pylyshyn & Storm, 1988), in which participants track one target presented with a like distractor by following the target’s spatiotemporal identity as the dots change position over time.

#### Stimuli

The dots were solid white circles that subtended 0.19° of visual angle and were presented within a 1.2° × 1.2° area at the center of, and superimposed over, the Gabors. At trial onset, the target and distractor dots were assigned unique random positions (no less than 0.31° apart) within the 1.2° × 1.2° area. At the start of the tracking interval, the target and distractor dots were independently assigned a motion direction vector, selected from 45°, 135°, 225°, and 315°. On each refresh, each dot had a 10% chance of being assigned a new vector, again selected from 45°, 135°, 225°, and 315°. In addition, whenever a dot neared the edge (within 0.3°), its vector was flipped. If the target and distractor dots neared each other (within 0.25°), the dots were assigned vectors to move in opposite directions.

#### Rate of feature change

At the start of the experiment, the dots moved at a rate of 0.035° per screen refresh (10 ms) in the easy condition, and at a rate of 0.047° per screen refresh in the difficult condition. Whenever participants made an error on single-task trials, the dot speed decreased by 0.0065° per refresh for subsequent blocks. In the hard condition, whenever accuracy was above/below 75%, the dot speed increased/decreased by 0.0065° per refresh.

## Experiment 1

Experiment 1 was designed to measure the amount of dual-task interference between auditory and visual attentive-tracking tasks. We asked participants to perform the tone-tracking task, the Gabor-tracking task, or both tasks simultaneously (see the individual task methods). We did not pair the tone-tracking and dot-tracking tasks, since both of these tasks involved monitoring the spatial position of stimuli over time and could therefore interfere purely because they both drew on a spatial code. Our tracking tasks always differed in the attended features.

We manipulated the difficulty of each task (easy or hard), and this created four distinct single-task conditions (Gabor–easy, Gabor–hard, tone–easy, tone–hard). There were eight dual-task conditions, differentiated by whether the response probe assessed the tone or the Gabor task, whether the probed task was easy or hard in difficulty, and whether the unprobed task was easy or hard in difficulty. Conditions were randomly intermixed within blocks. Fourteen individuals (seven female, seven male) between the ages of 18 and 29 years (mean age 21.1) gave informed consent to participate.

### Results and discussion

To provide the least noisy estimate of the condition means (lower variability), analyses were conducted using all intermixed blocks (excluding only the first two single-task blocks). The main conclusions were unchanged if only the second session was analyzed. Note that, to avoid confusion, we use the term *difficulty* to refer to the task demands of the task that performance was probed on, and the term *load* to reference the task demands of the task that was not probed (secondary task). This allowed for an assessment of task performance for both levels of task difficulty across three levels of secondary task load: single-task, low load (easy), and high load (hard).

#### Single-task performance

**Fig. 2:**
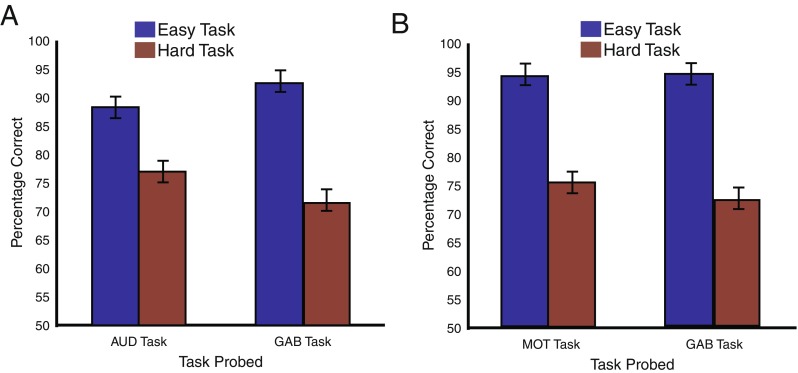
Single-task accuracy for the multimodal (**a**) and unimodal (**b**) experiments, as a function of task difficulty and task. Error bars represent within-subjects errors of the main effect of task

Percentages correct for the single-task conditions by task and difficulty are shown in Fig. 2a. To compare single-task performance across tasks, the percentages correct for single-task trials were entered into a 2 × 2 analysis of variance (ANOVA) with the factors difficulty (easy, hard) and task (Gabor, tone). This revealed a main effect of difficulty [*F*(1, 13) = 50.83, *p* < .001], no main effect of task [*F*(1, 13) = 0.02, *p* = .90], and an interaction [*F*(1, 13) = 11.22, *p* < .01].

The results show that the difficulty manipulation was successful—hard trials had significantly lower performance, suggesting that performance for those tasks was more demanding. Also, the fact that no main effect of task was observed suggests that the tasks may have been equivalent in their demands. However, the interaction between task and difficulty suggests that the difficulty titration did not work equivalently for each task. Indeed, the effect of difficulty in single-task conditions was smaller for the tone task than for the Gabor task (*p* < .05; *t* test comparing changes in performance across tasks)*.* Difficulty for the tone task was adjusted by manipulating the number of transitions. As we explained in the General Method section, increasing the number of transition cycles led to a concomitant increase in the alternation rate between tones. Studies have shown that the ability to perceptually segregate streams improves as the alternation rate increases (Bregman & Campbell, 1971). Although this effect occurs largely outside of the range of alternations in the present study (it is typically found at slower alternation rates), it is possible that the effect may have reduced the magnitude of the auditory difficulty manipulation. As a consequence, the present study may actually underestimate the dual-task costs when the effect of auditory load on the visual task performance is considered.

#### Comparison of dual-task versus single-task conditions

**Fig. 3:**
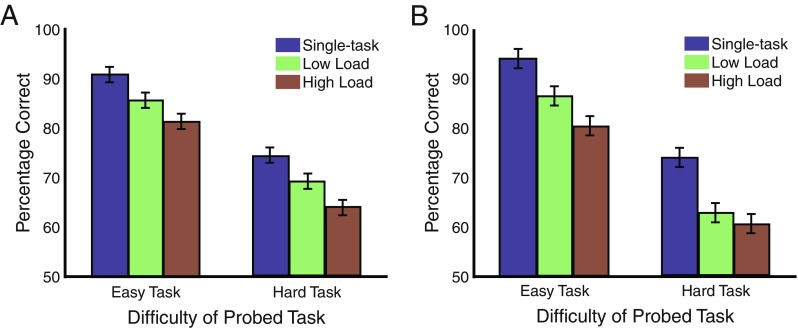
Task accuracy for the multimodal (**a**) and unimodal (**b**) experiments, as a function of task difficulty and secondary task load. Error bars represent within-subjects errors of the main effect of load

Figure 3a shows the percentages of correct target identification as a function of probed task difficulty and secondary task load. Because dual-task costs could lead to worse performance on either the tone- or the Gabor-tracking task, depending on how participants choose to distribute limited resources, these costs may not be meaningful when considered separately for each task (see Fougnie & Marois, 2006). For this reason, in the analysis below we collapsed across the tasks. The individual task data can be found in the supplemental materials.

The percentages correct were entered into a 2 × 3 ANOVA with the factors difficulty (easy, hard) and secondary task load (single-task, low, high). The results showed a main effect of difficulty [*F*(1, 13) = 90.05, *p* < .001], with worse performance for hard trials. Additionally, we observed a main effect of secondary task load [*F*(2, 13) = 20.97, *p* < .001], but no interaction [*F*(2, 13) = 0.10, *p* = .90].

These results show, in contrast to several previous studies (Alais et al., 2006; Duncan et al., 1997; Larsen et al., 2003; Martens et al., 2010; Soto-Faraco & Spence, 2002; Treisman & Davies, 1973), that visual and auditory perceptual attention tasks can substantively interfere with each other. However, it is not clear from the results above what is the source of this interference. One possibility is that it results from the demands to coordinate dual-tasking (i.e., concurrence costs). Another possibility, which is not mutually exclusive of the first, is that it reflects the tracking demands of each task (load-dependent dual-task costs). To address the first possibility, we ran a 2 × 2 ANOVA with the factors difficulty (easy, hard) and task number (single task, low-load dual task). This analysis revealed main effects of difficulty [*F*(1, 13) = 67.93, *p* < .001] and task number [*F*(1, 13) = 13.05, *p* = .003], but no interaction [*F*(1, 13) = 0.02, *p* = .89], suggesting that performing a concurrent tracking task, even an easy one, impairs performance of the primary tracking task. To address the second possibility, we ran a 2 × 2 ANOVA with the factors difficulty (easy, hard) and secondary task load (low, high). That analysis revealed main effects of difficulty [*F*(1, 13) = 68.39, *p* < .001] and secondary task load [*F*(1, 13) = 8.11, *p* < .05], but no interaction [*F*(1, 13) = 0.08, *p* = .76]. This is strong evidence for competition between auditory and visual tracking loads. Note that the single- and dual-task conditions were perceptually equivalent during the tracking phase, indicating that differences in low-level sensory stimulation cannot explain these results. We therefore conclude that interference between an auditory and a visual tracking task is caused by both the demands to coordinate the execution of the two tasks and the attentional demands of simultaneously tracking auditory and visual objects.

## Experiment 2

Experiment 1 showed cross-modal interference in tasks that required tracking of perceptual information over time, in contrast to accounts that suggest that auditory and visual attention have independent attention capacities (Alais et al., 2006; Duncan et al., 1997). The goal of Experiment 2 was to allow for a comparison of the costs observed between two tracking tasks in different modalities and two tasks that shared a modality, in order to assess the degree to which tracking draws on supramodal and modality-specific sources. In Experiment 2 we paired two visual tracking tasks. Specifically, Experiment 2 required 14 new participants (nine females, five males; ages 18 to 26 years, mean age 19.9) to perform the Gabor-tracking task, the dot-tracking task, or both simultaneously (see Fig. 1). In other respects, this experiment was the same as Experiment 1.

### Results and discussion

#### Single-task performance

Percentages correct for the single-task conditions, separated by task and difficulty, are shown in Fig. 2b. To compare single-task performance across the tasks, the percentages correct for single-task trials were entered into a 2 × 2 ANOVA with the factors difficulty (easy, hard) and task (Gabor, tone). This revealed a main effect of difficulty [*F*(1, 13) = 177.27, *p* < .001], no main effect of task [*F*(1, 13) = 0.52, *p* = .48], and no interaction [*F*(1, 13) = 0.38, *p* = .55]. Thus, the two tasks were equivalently difficult for participants across both the easy and hard difficulty conditions.

#### Comparison of dual-task versus single-task conditions

Figure 3b shows percentages correct as a function of probed task difficulty and secondary task load, averaged across the tasks. As in Experiment 1, dual-task costs are collapsed across tasks, though the results for each task separately can be found in the supplemental material.

The percentages correct were entered into a 2 × 3 ANOVA with the factors difficulty (easy, hard) and load (single-task, low, high). The results showed a main effect of difficulty [*F*(1, 13) = 118.36, *p* < .001], with worse performance for hard trials. Additionally, there was a main effect of secondary task load [*F*(2, 13) = 26.73, *p* < .001], but no interaction [*F*(2, 13) = 0.72, *p* = .50]. Additional analyses were performed to assess the concurrence and load-dependent costs. A 2 × 2 ANOVA with the factors difficulty (easy, hard) and task number (single task, low-load dual task) revealed main effects of difficulty [*F*(1, 13) = 87.86, *p* < .001] and task number [*F*(1, 13) = 30.47, *p* < .001], but no interaction [*F*(1, 13) = 2.34, *p* = .15], suggesting that performing two concurrent visual tracking tasks causes the tasks to interfere with one another. A second 2 × 2 ANOVA with the factors difficulty (easy, hard) and secondary task load (low, high) showed main effects of difficulty [*F*(1, 13) = 74.98, *p* < .001] and secondary task load [*F*(1, 13) = 5.20, *p* < .05], but no interaction [*F*(1, 13) = 0.79, *p* = .39]. This result suggests that interference between two visual tracking tasks is load-dependent.

The fact that the present study showed strong, load-dependent costs between two visual tracking tasks is not surprising and is consistent with previous studies (Lee et al., 1999; Pastukhov, Fischer, & Braun, 2009). However, this study went beyond the previous experiments by also manipulating attentional load. The results show that dual-task costs increased with increased load. Thus, a novel contribution of this study is to suggest that the previous evidence of interference between two different visual tasks was not due simply to the executive demands required to coordinate two tasks.

### Comparison of costs in Experiments 1 and 2

Dual-task costs were compared across Experiments 1 and 2, to test whether there were differences in performance between the multimodal and unimodal task pairings. Task accuracy was entered into a between-subjects 2 × 2 × 3 ANOVA with the factors experiment (multimodal, unimodal), difficulty (easy, hard), and secondary task load (single-task, low, high). This revealed a main effect of difficulty [*F*(1, 26) = 208.31, *p* < .001] a main effect of secondary task load [*F*(2, 52) = 47.03, *p* < .001], but no main effect of experiment [*F*(1, 26) = 0.63, *p* < .43]. The interaction between experiment and secondary task load was also not significant [*F*(2, 52) = 2.05. *p* = .14]. No other interactions were significant (all *p*s > .1).

These initial results suggest that the intra- and cross-modal costs of dual-tasking may be equivalent. To further identify the origin of these costs, we then broke down the effect of modality pairing (intra- vs. cross-modal) on the two sources of dual-task costs: concurrence costs (the decrement in performance between single-task conditions and dual-task conditions with low secondary task load) and load-dependent costs (an additional decrease in performance due to increased secondary task load). To compare concurrence costs, participants’ accuracy percentages for the low-load dual-task conditions were subtracted from their performance in the single-task conditions (separately for each level of task difficulty). These costs were entered into a between-subjects 2 × 2 ANOVA with the factors experiment (multimodal, unimodal) and difficulty (easy, hard). The ANOVA showed a marginal effect of experiment [*F*(1, 26) = 4.21, *p* = .054], with greater concurrence costs in the unimodal condition, but no main effect of difficulty [*F*(1, 26) = 1.06, *p* = .31] and no interaction between experiment and difficulty [*F*(1, 26) = 0.64, *p* = .43]. These results suggest that adding a secondary tracking task incurred marginally larger costs in the unimodal than in the multimodal conditions.

To compare load-dependent costs, participants’ accuracy percentages for the high-load dual-task conditions were subtracted from their performance in the low-load dual-task conditions and entered into a between-subjects 2 × 2 ANOVA with the factors experiment (multimodal, unimodal) and difficulty (easy, hard). The ANOVA revealed no main effect of experiment [*F*(1, 26) = 0.05, *p* = .82], no main effect of difficulty [*F*(1, 26) = 0.31, *p* = .56], and no interaction between experiment and difficulty [*F*(1, 26) = 0.80, *p* = .38]. The lack of an effect of experiment suggests no difference between the experiments due to increasing task load. However, it is important to note that this is a null effect, and therefore has limited interpretive power. To bolster our finding, we quantified the amount of evidence in favor of the null hypothesis by using Bayes factor analysis (Rouder, Speckman, Sun, Morey, & Iverson, 2009). We calculated the Bayes factor by assessing the overall concurrence costs (averaging across task difficulty) for the two experiments and comparing them in a two-sample *t* test. The resulting Bayes factor of 2.88 (*t* statistic of 0.20) suggests that the null hypothesis was about three times more probably than the alternative hypothesis. This is marginal, not decisive, evidence in favor of the null.

Differences in performance between the unimodal and multimodal experiments were revealed when the dual-tasks costs were separated into concurrence and load-dependent costs. Specifically, whereas the load-dependent costs were found to be similar across both experiments, the concurrence costs were marginally higher in the unimodal than in the multimodal experiments. This finding indicates that although auditory and visual tracking tasks do interfere, they do not interfere to the same degree as two tracking tasks from the same modality.

Importantly, however, an auditory and a visual tracking task did not simply interfere with one another because of the demands to concurrently perform two tasks at once. The load manipulations clearly indicated that the interference between a visual and an auditory tracking task was load-dependent. Moreover, the finding that the load-dependent costs did not differ between multimodal (Exp. 1) and unimodal (Exp. 2) conditions raises the intriguing possibility that the increased perceptual load of tracking draws only on domain-general sources of processing. Of course, the latter conclusion rests on accepting the null hypothesis, even though it was fortified by the Bayes factor analysis. Furthermore, in Experiment 2, performance in the hard difficulty condition was already close to chance under low load, and therefore the magnitude of the dual-task costs may have been underestimated. However, a comparison of the load-dependent costs in the easy difficulty conditions of Experiments 1 and 2 showed no difference (*p* = .60), thereby providing some evidence for the equivalency of unimodal and cross-modal load costs in a condition that was not subject to potential floor effects in performance.

## General discussion

Many common activities, such as navigating a busy intersection, playing a team sport, or listening to the symphony, require attending to and following individual elements as they change over time. A large body of research has detailed limitations in the number of objects that can be simultaneously attended and tracked in visual displays (Alvarez & Cavanagh, 2005; Alvarez & Franconeri, 2007; Blaser et al., 2000; Fehd & Seiffert, 2008; Franconeri, Jonathan, & Scimeca, 2010; Pylyshyn & Storm, 1988; Scholl & Pylyshyn, 1999; Sears & Pylyshyn, 2000; Yantis, 1992). However, although tracking has been studied extensively in the visual domain, little is known about tracking in other modalities and about the extent to which tracking is limited by modal or supramodal mechanisms. To address this issue, we developed a novel auditory tracking task in which participants tracked one of two auditory streams by following a target’s pitch and stereo position over time. We used this auditory tracking task and two distinct visual tracking tasks to measure the interference between tasks that either shared or differed in their perceptual modality. To our knowledge, this was the first study to use an auditory tracking task and combine it with a visual tracking task to examine the domain specificity of tracking capacity.

In Experiment 1 we paired an auditory tone-tracking task with a visual Gabor-tracking task (multimodal pairing). In Experiment 2 we paired a visual dot-tracking task with a visual Gabor-tracking task (unimodal pairing). For both the multimodal and unimodal task pairings, we not only compared performance between the single- and dual-task conditions, but also manipulated task difficulty in order to measure the degree to which the interference between the tasks scaled with task difficulty.

In Experiment 1 we found significant dual-task costs between the auditory and visual tracking tasks, and these costs increased with increased tracking load. These results demonstrate that auditory and visual tracking must draw on a common resource. The observation of load-dependent dual-task costs rules out the possibility that interference across the modalities was simply due to the executive demands of preparing for and executing two tasks (De Jong & Sweet, 1994). In addition, our findings cannot be explained by perceptual interference since the perceptual demands during the tracking phase were equated between single- and dual-task conditions.

We conducted Experiment 2 to measure the interference between two visual tracking tasks, with the goal of comparing these costs to those observed in the multimodal pairing in Experiment 1. This comparison suggested a modality-specific source of visual tracking capacity above and beyond the multimodal capacity limits identified in Experiment 1. Interestingly, however, that visual modality-specific source of interference emerged only when comparing single-task performance to dual-task conditions with low load (concurrence costs). These modality-specific concurrence costs may reflect a shorter “functional distance” (Kinsbourne & Hicks, 1978) for tasks that share a perceptual modality. That is, the neural substrates of the visual tracking tasks may have overlapped to a greater extent (relative to the auditory and visual pairing), causing stronger interference or competition.

Importantly, we found no evidence for greater interference in the unimodal condition when task load was increased. This unexpected result may imply that increasing the tracking load draws entirely on domain-general sources of capacity. However, since this would require accepting the null hypothesis, further experimentation will be necessary to substantiate this finding. It is also important to point out that our findings are based on one specific manipulation of tracking load, namely the rate of change in the features being tracked. It remains to be determined whether similar results would also apply to different forms of tracking load, particularly with regard to the number of items to be tracked. Although there is evidence that the quantity of tracked items and the rate of change in tracked items load on the same tracking resource (Alvarez & Franconeri, 2007; Franconeri, Lin, Pylyshyn, Fisher, & Enns, 2008), it is still possible that these types of load may differentially affect tracking (Shim, Alvarez, Vickery, & Jiang, 2010). Experimental inquiry into this issue must await significant modification of our present auditory tracking task, however, since it was not possible with the present design to increase the number of target or distractors streams without making it impossible for participants to segregate the tones into coherent streams.

Many theories differentiate between perceptual attention (attending to perceptual inputs) and central attention (selection at postperceptual stages of processing, such as response selection) (Chun et al., 2011; Duncan et al., 1997; Johnston, McCann, & Remington, 1995; Luck & Vecera, 2002; Pashler, 1989; Posner & Boies, 1971). Whereas central attention is considered amodal, perceptual attention is regarded as modality-specific (Tamber-Rosenau & Marois, 2016). With respect to that framework, mounting evidence suggests that classifying multiple-object tracking (MOT) paradigms as perceptual attention tasks (Alvarez & Cavanagh, 2005; Scholl, 2001; Sears & Pylyshyn, 2000) may not be warranted. Not only did we find here that MOT largely draw on a central, capacity-limited resource, several other studies have shown that central “executive” tasks—such as backward counting, word generation, and response selection—interfere with attentive tracking (Allen et al., 2006; Kunar et al., 2008; Tombu & Seiffert, 2008). We suggest that tracking tasks draw on central attention because they require adaptive control and updating of attended information, as the featural information (spatiotemporal identity) that differentiates targets from distractors changes over the course of tracking in an unpredictable manner.

This account can also explain why our findings are inconsistent with theories suggesting independent attention capacities across modalities (Alais et al., 2006; Arrighi, Lunardi, & Burr, 2011; Chun et al., 2011; Duncan et al., 1997; Pashler, 1989). Evidence for the latter view draws largely from studies that have required monitoring of multiple sources of information in order to perform target detection or discrimination. Such studies have shown that although participants are unable to simultaneously monitor two sources that share a modality (Bonnel & Miller, 1994; Lee et al., 1999; Norman & Bobrow, 1975; Sperling & Dosher, 1986), they can monitor auditory and visual sources as efficiently as either single modality (Alais et al., 2006; Duncan et al., 1997; Larsen et al., 2003; Martens et al., 2009; Martens et al., 2010; Soto-Faraco & Spence, 2002; Treisman & Davies, 1973). We surmise that the difference in findings between these studies and ours is that the tasks in the other studies did not require participants to dynamically adjust their attentional set within the context of a trial. Since the to-be-attended information was known to the participants before target onset, detection or identification may have been possible by simply setting up target templates in working memory (Duncan & Humphreys, 1989). Once established, these templates might not require any executive processes for their simple maintenance, in contrast to the constant template readjustment required in tracking tasks. According to this scheme, these tasks would draw purely on perceptual attention, thus explaining why they would not reveal dual-task interference across modalities.

The substantial interference between auditory and visual tracking shown in the present study points to limits in the benefits of multisensory perceptual input. This insight is important, not just for theory, but also for human-factors science. To reduce accidents stemming from overloaded perceptual processing, there have been calls for multisensory approaches to the design of airplane cockpits and automobile dashboards (Brickman, Hettinger, & Haas, 2000; Ho & Spence, 2008), since this could allow for parallel processing of task-relevant sensory information. The present findings suggest that multisensory sources of information may not provide the expected boost in performance, at least when competing, not congruent, sources of information need to be attentively tracked over time. Thus, important consideration should be given as to how to minimize online demands in order to maximize the benefits of multisensory processing.

## Electronic supplementary material

ESM 1 (GIF 85 kb)

High resolution image (EPS 3247 kb)

ESM 2 (GIF 20 kb)

High resolution image (EPS 1275 kb)

ESM 3 (GIF 60 kb)

High resolution image (EPS 1476 kb)
