# Supplementary figures and images for: A common source of attention for auditory and visual tracking
Source: Atten Percept Psychophys. 2018 May 1;80(6):1571–83. doi: 10.3758/s13414-018-1524-9 (PMC6061001; doi:10.3758/s13414-018-1524-9)

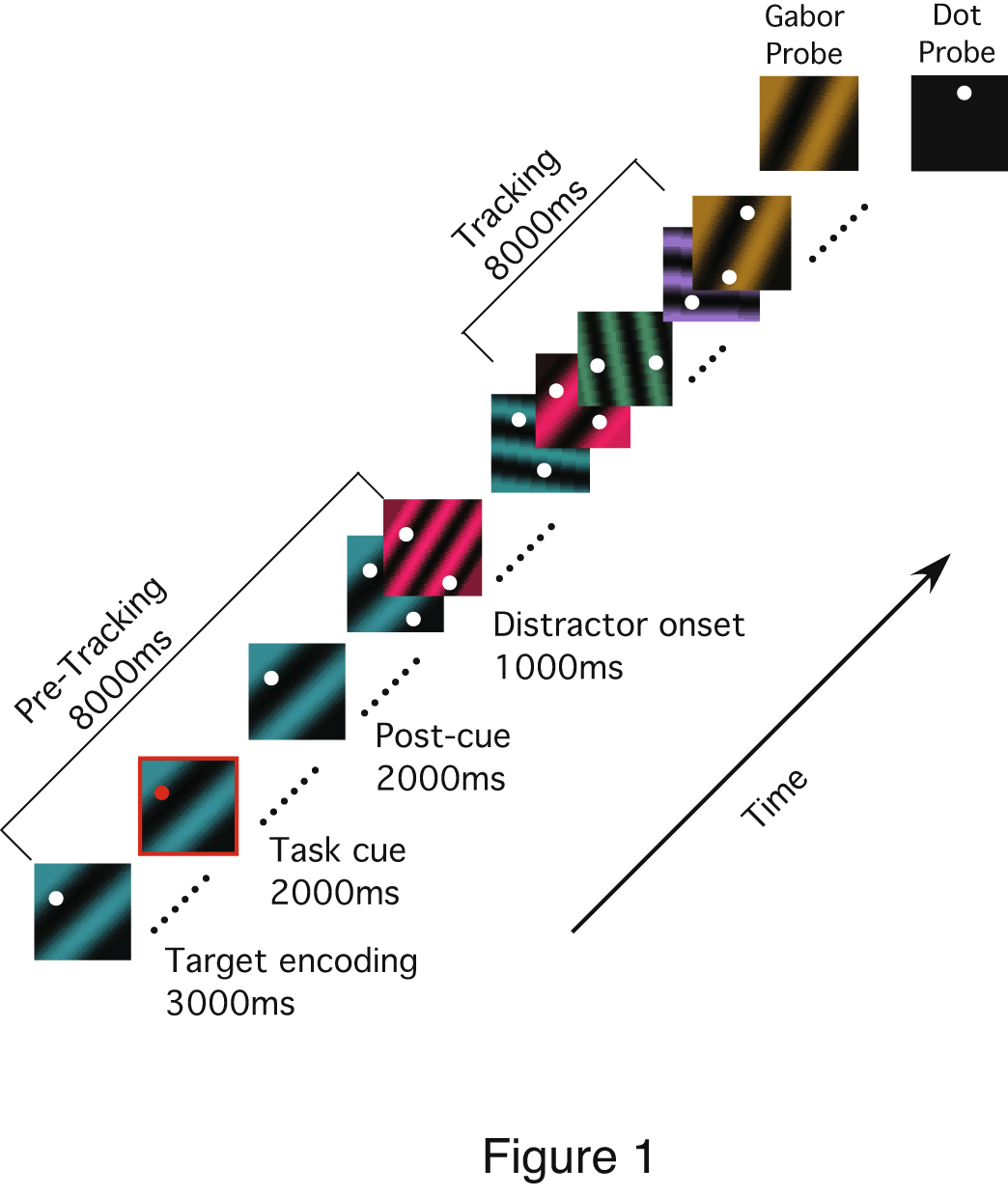

Supplement: Supplementary file 1 — (GIF 85 kb) [file 13414_2018_1524_Fig4_ESM.gif]

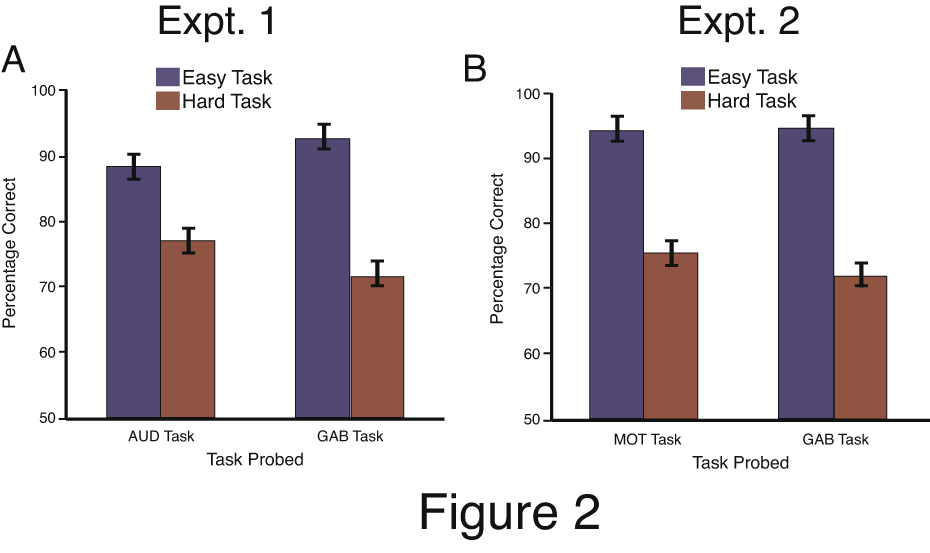

Supplement: Supplementary file 3 — (GIF 20 kb) [file 13414_2018_1524_Fig5_ESM.gif]

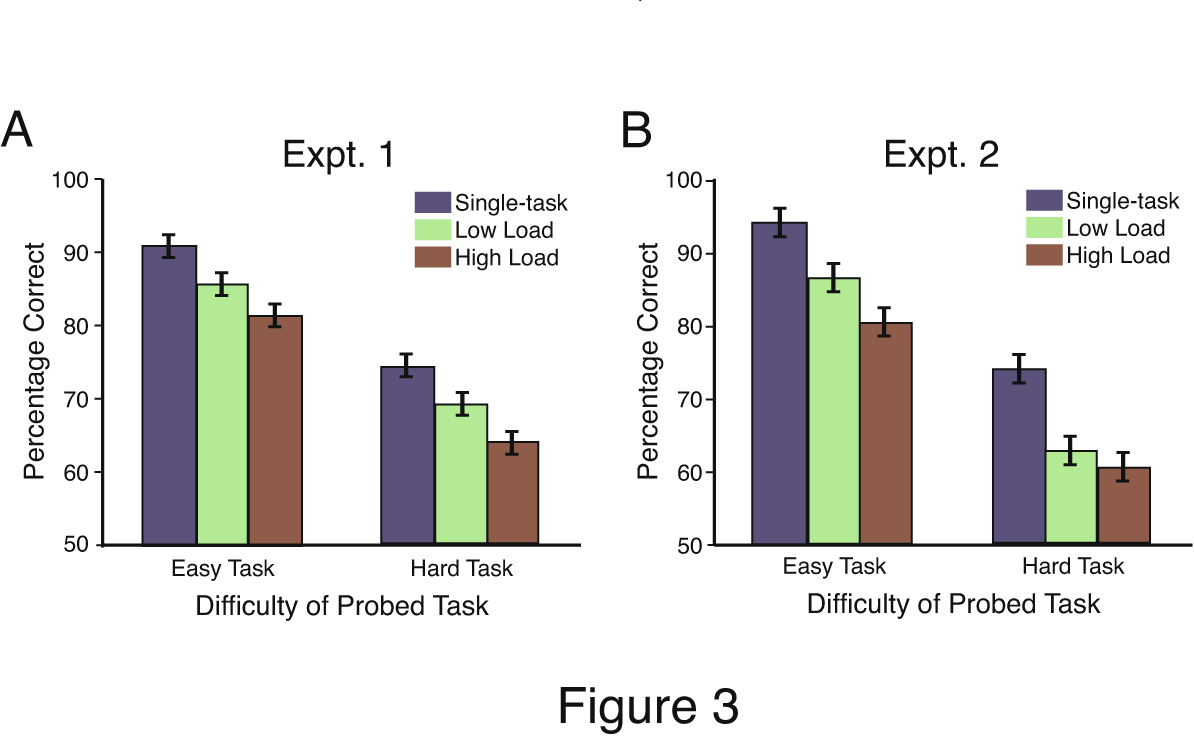

Supplement: Supplementary file 5 — (GIF 60 kb) [file 13414_2018_1524_Fig6_ESM.gif]
